# Exposure to Ambient Air Particles Increases the Risk of Mental Disorder: Findings from a Natural Experiment in Beijing

**DOI:** 10.3390/ijerph15010160

**Published:** 2018-01-19

**Authors:** Zhen Jia, Yongjie Wei, Xiaoqian Li, Lixin Yang, Huijie Liu, Chen Guo, Lulu Zhang, Nannan Li, Shaojuan Guo, Yan Qian, Zhigang Li

**Affiliations:** 1 Beijing Institutes of Life Science, Chinese Academy of Sciences, Beijing 100000, China; jiazhen15@mails.ucas.ac.cn; 2 Laboratory of Environmental Criteria and Risk Assessment and Environmental Standards Institute, Chinese Research Academy of Environmental Sciences, Beijing 100000, China; lixiaoqian@craes.org.cn (X.L.); yanglx@craes.org.cn (L.Y.); marchjie1990@163.com (H.L.); chenpreteen@sina.com (C.G.); zhang_lulu@yeah.net (L.Z.); linannqinian@163.com (N.L.); guosj@craes.org.cn (S.G.); qianyan@craes.org.cn (Y.Q.)

**Keywords:** PM_2.5_, mental disorder, glucocorticoid receptors, glucocorticoid, inflammation

## Abstract

Epidemiology studies indicated that air pollution has been associated with adverse neurological effects in human. Moreover, the secretion of glucocorticoid (GC) affects the mood regulation, and the negative feedback of hippocampal glucocorticoid receptors (GR) inhibits the GC secretion. Meanwhile, the over secretion of GC can interfere the immune system and induce neurotoxicity. In the present study, the human test showed that the secretion of the cortisol in plasma was elevated after exposure in heavy air pollution. In the mouse model, we found that breathing the highly polluted air resulted in the negative responses of the mood-related behavioral tests and morphology of hippocampus, as well as the over secretion of GC in plasma, down regulation of GR, and up-regulation of cytokine and chemokine in the hippocampus. When considering the interrelated trends between the hippocampal GR, inflammatory factors, and plasmatic GC, we speculated that PM_2.5_ exposure could lead to the increased secretion of GC in plasma by decreasing the expression of GR in hippocampus, which activated the inflammation response, and finally induced neurotoxicity, suggesting that PM_2.5_ exposure negatively affects mood regulation. When combined with the results of the human test, it indicated that exposure to ambient air particles increased the risk of mental disorder.

## 1. Introduction

In recent years, when compared with the past, the mental illnesses are increasing in China, such as depression, autism, anxiety, and other related diseases, especially in children. By now, although the real causes are not so clear, the biological, the psychological, and the social environment should all have the possibility to be involved in the pathogenesis of these diseases. Among them, the air pollution is also imagined as one of the factors. Epidemiology and toxicology studies showed that PM_2.5_ could induce neurodevelopmental disorders, which can cause many cognition-related diseases, including autism-spectrum disorders (ASD) [1,2,3], schizophrenia, anxiety-spectrum disorders, and depression [4,5,6,7]. Population-based researches showed that exposure to particulate matter (PM) from prenatal to first postnatal year led to higher risk of ASD [1,3]. In childhood, even in elderly stages, severe urban air pollution impaired cognitive functions and caused white matter abnormalities [7,8]. Prenatal and childhood traffic-related pollution and air particles exposure affected cognitive development [9,10,11], and correspondingly, the reduction of air pollution could result in beneficial effects on children cognition [11]. The toxicology works also reported that exposures to PM were associated with cognitive impairment. After being exposed to PM, the mice exhibited more depression-like responses and weaknesses of the spatial memory, as well as the decrease of the density and branching in the hippocampus [12]. Thus, it is important to evaluate biological indicators of mental changes after exposure to PM, in order to identify risk factors of health and disease.

The glucocorticoid (GC) was sensitive in mood disorders [13].The key system in regulating GC secretion was the hypothalamic-pituitary-adrenal (HPA) axis, of which the cortisol is the main hormone in humans [14,15]. In both remitted and currently depressed patients, the cortisol levels were higher when compared with healthy people [16]. In patients with acute depression, a 25% higher of cortisol was observed [16,17]. The patients with history of depression or anxiety also had high cortisol level [18]. Therefore, we suppose that the possibility of mental disease/symptom can be reflected by the concentration of GC in plasma. When considering the lack of clarity of the biological indicator of PM exposure on the mood related behaviors, in the present study, we examined the neurobehavioral regulation related cortisol in plasma of human volunteers after ambient air particles exposure. Furthermore, in order to verify the influence of PM exposure on mood regulation, a series of behavioral assays experiments and detection of molecular biomarkers were also conducted with the mouse model.

## 2. Materials and Methods

### 2.1. Ethics

All human being and animal experimentations were conducted in compliance with the guidelines of ethical human being and animal research. The study protocol was approved by the Medicine Animal Care and Use Committee at Aviation General Hospital before commencement of experiments.

### 2.2. Exposure Protocol

The study population consisted of 12 non-smoking male adults, without occupationally exposed to air pollutants and no previous medical history. We chose the day with high pollution level (276.9 ± 50.4 μg/m^3^) as exposure day (21–26 February 2014) and low pollution level (47.6 ± 37.3 μg/m^3^) as control day (9–20 December 2013). After high and low concentration of PM_2.5_ exposure, they were phlebotomized and separated the plasma for testing.

To the animal model, we used the same set of chambers as before [19]. Briefly, the two stainless steel chambers (1.2 m × 0.8 m × 1.2 m) for animal exposure were paralleled at the clean-level air-conditioned room, with the temperature of 24 ± 1 °C inside. The two chambers were connected to the 25–35 outside with stainless steel pipelines. The real-world ambient air was introduced to the chambers by induced draft fan. The only difference between the two chambers is at the inlet of PM filtered chamber, there is a high-efficiency particulate air filter (HEPA) unit. The filtration efficiency of the HEPA unit was 98.99% ± 0.86% for particles larger than 2.5 μm in aerodynamic diameter and 70.61% ± 19.34% for PM_2.5_.

The C57 BL/6 J male mice were used in our experiment. Eight weeks old mice were randomly divided into two groups. 10 mice entered the unfiltered chamber, and 10 mice entered the filtered chamber. Mice lived in these chambers naturally under a 12 h light/12 h dark cycle and were fed a normal chow diet. During the experiment period of mice, PM_2.5_ concentrations were 91.3 ± 84.8 μg/m^3^ in the unfiltered chamber and 17.9 ± 7.8μg/m^3^ in the filtered chamber. After 140 days exposure (7 December 2013 to 15 March 2014), we did a series of behavioral experiments for testing the responses of animals and harvested the hippocampus and blood of mice.

### 2.3. Determination of Plasma Glucocorticoid (GC) Levels

Blood of human beings and mice was drawn into chilled vacuum blood collection tubes containing K2EDTA. The supernatant plasma of each sample was collected after centrifuge for 30 min at 3000× *g* at 2–8 °C and stored at −80 °C until use. Plasma GC level was measured by cortisol (in human) and corticosterone (in mice) immunoassay (Research and Diagnostic Systems Inc., Minneapolis, MN, USA), according to the manufacturer’s recommended protocol.

### 2.4. Mouse Behavioral Assays

#### 2.4.1. Open Field Test (OFT)

The equipment for the OFT was a square arena (30 cm × 30 cm × 40 cm), illuminated by a set of 4 light beams arrays overhead in the horizontal *X* and *Y* axes. Mice were allowed to freely explore for 30-min after they were placed in the center of the arena. The tracks of mice were analyzed by a video-tracking system. In the OFT, the area around 30 cm of the center (18 cm × 18 cm) was defined as the central zone. The time that mice spent in the center zone were recorded.

#### 2.4.2. Elevated Plus Maze Test (EPMT)

The apparatus used for the elevated plus maze test comprised two open arms (30 cm × 7 cm × 0.5 cm) across from each other and perpendicular to two closed arms (30 cm × 7 cm × 16 cm) with a center platform (7 cm × 7 cm). The closed arms have a high wall (16 cm) to enclose the arm. The apparatus was elevated to a height of 100 cm above the floor. A mouse is placed in the center area, and allowed to freely explore the apparatus for 5 min. All of the processes were recorded by using a video camera fixed above the EPMT. The percentage of the time the mouse spent in the open arms vs. the total time is served as the index of anxiety-like behavior.

#### 2.4.3. Light/Dark Box Test (L/DBT)

Briefly, a dark (2 lux) and a light box (300 lux) were separated to equal sizes by a partition with a small opening area at floor level. The mice were allowed to move freely between the two boxes for 5 min with door open and videotaped. The percentage of the time the mouse spent in the light chamber vs the total time was served as the index of anxiety-like behavior.

#### 2.4.4. Forced Swim Test (FST)

In the FST, a mouse was slowly placed in a cylindrical tank (18 cm in diameter) filled with 25 cm depth of water at room temperature (23–27 °C). On the pre-test day, the mouse was placed in the water for 15 min. On the test day, the mice were placed in water for 6 min and their behaviors were recorded by a high-resolution video camera. The mobility in the FST was defined as any movement other than those necessary to balance the body and keep the head above the water. The immobility time was calculated by the subtraction of 360 s to the total amount of mobility time.

#### 2.4.5. Social Interaction Test (SIT)

Social interaction behavior was assessed by social interaction test (SIT) in an open-field apparatus (30 cm × 30 cm) for 2.5 min. A mouse was placed into a small empty mesh cage (7 cm × 4 cm) on one wall (target absent). The time that the experimental mouse spent in the interaction zone (IZ), around the mesh cage (10 cm × 18 cm), was defined as the interaction duration.

### 2.5. Hippocampus Morphometric Analysis

The completely cut-down hippocampus were fixed in 10% formalin and embedded in paraffin for making hematoxylin and eosin (HE) stained slides, performing morphometric analysis. These slides were analyzed by an optical microscope with a digital camera (200×).

### 2.6. RNA-Sequence (RNA-Seq) and Bioinformatics Processing of the Data

Total RNA was extracted from the hippocampus of unfiltered and filtered mice by using the RNeasy Mini Kit (QIAGEN Str., Hilden, Germany). The RNA quality was assessed by using the Bioanalyser 2100 RNA 6000 Nano Kit (Agilent Technologies, Santa Clara, CA, USA). Second-generation RNA sequencing (RNA-Seq) was used to analyze the genome-wide gene expression profiling in the hippocampus. RNA-Seq was analyzed by a commercial company (BGI, Shenzhen, China). The gene expression results are presented as the log2 ratio of data from the unfiltered air group to the data from the filtered air group. The genes, which p-values and false discovery rate (FDR) values below 0.05 were considered as differentially expressed genes between unfiltered and filtered groups.

### 2.7. Real-Time PCR (Polymerase Chain Reaction)

Real-time PCR validation was conducted using the Maxima^®^ SYBR^®^ Green qPCR Master Mix kit (CWBIOtech, Taipei, Taiwan) according to the manufacturer’s instructions, in an ABI Prism 7500 Sequence Detection System 288 (Applied Biosystems Inc., Foster City, CA, USA), with the following program: 95 °C for 10 min; 45 cycles of 95 °C for 25 s, 60 °C for 1 min; and, 72 °C for 10 min. Table S4 showed the primer of selected genes. Transcript levels were normalized to Gapdh levels.

### 2.8. Western Blotting

To examine the expression level of glucocorticoid receptors (GR) (Cat. #3660, Cell signaling), hippocampal lysates (each 10 μg) were separated by SDS-PAGE (Sodium Dodecyl Sulfate Polyacrylamide Gel Electrophoresis) (Solarbio, Beijing, China) for 2 h at 100V and transferred to a PVDF (Polyvinylidene Fluoride) membrane at 230 mA for 1 h. All of the membranes were blocked with 5% BSA (Bovine serum albumin) in 1× TBS (Tris-buffered saline) containing 0.1% Tween 20 for 1 h and probed with primary and secondary antibodies at 4 °C overnight. 

### 2.9. Statistical Analyses

Results are presented as means ± standard error of the means. Statistical tests were performed using Student’s *t*-test after the analysis of homogeneity of variance and normal distribution. All statistical analyses were performed using GraphPad Prism (Graphpad Software, Inc., La Jolla, CA, USA).

## 3. Results

### 3.1. Increased GC Level of Plasma by Exposure to PM_2.5_ in Both Human and Mice

**Figure 1:**
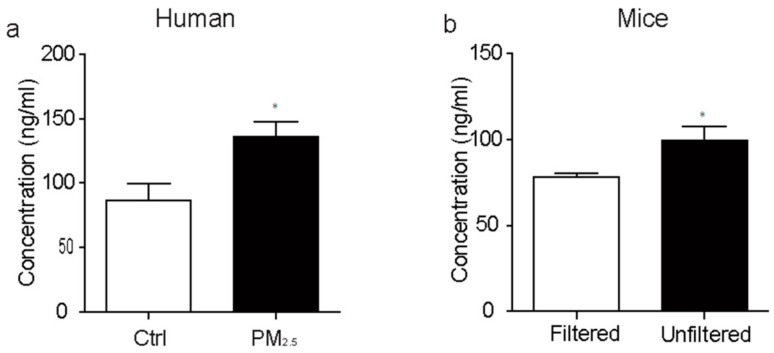
Exposure to ambient air particles up-regulate the concentration of plasma glucocorticoid in both human (**a**) *p* < 0.05, t22 = 2.085, and mice (**b**) *p* < 0.05, t18 = 2.608; * *p* < 0.05.

For human being volunteers, we found that exposure to PM_2.5_ increased the plasma cortisol levels in human (Figure 1a). Consistent with the results of human, we also found that exposure to PM_2.5_ significantly increased plasma GC levels in mice (Figure 1b). The mood disorders patients have higher mean cortisol levels [13], combined with our result, suggesting the exposure to ambient air particles increases the risk of mental disorder.

### 3.2. Mood-Related Behaviors are Impaired by Exposure to PM_2.5_ in Mice

**Figure 2:**
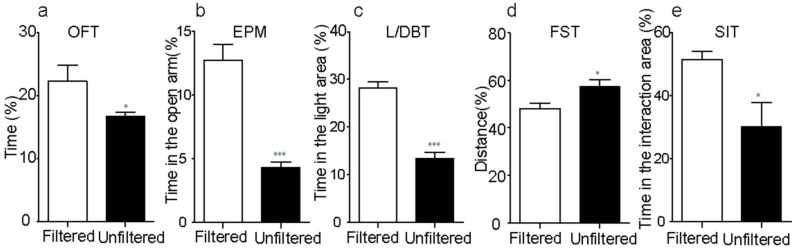
Exposure to ambient air particles provokes mood-related behaviors disorder. (**a**–**c**) Unfiltered mice spend less time in the center area of the open field arena in the open field test (OFT) (*p* < 0.05, t18 = 2.161), the open arm of the elevated plus maze test (EPM) (*p* < 0.001, t18 = 6.333) and the light side of the light-dark box test (L/DBT) (*p* < 0.001, t18 = 7.776). (**d**) Unfiltered mice displayed significantly more immobility than filtered mice in forced swim test (FST) (*p* < 0.01, t18 = 3.688). (**e**) The time in the interaction area of unfiltered mice was less than that of filtered mice in social interaction test (SIT) (*p* < 0.05, t18 = 2.583). * *p* < 0.05; *** *p* < 0.001.

To examine the effects of exposure to PM_2.5_ on mood-related behaviors, adult mice were exposed to real-world PM_2.5_ for 140 days in exposure chambers. First of all, for detecting the responses of anxiety-related behavior, we performed the OFT, L/DBT, and EPMT experiments. When compared with filtered group, the unfiltered mice travelled less time in the center arena of OFT (Figure 2a) and spent less time in both the open arm of EPMT (Figure 2b) and the light side of the L/DBT (Figure 2c). Furthermore, we also investigated the influence of exposure to PM_2.5_ on SIT and FST, which reflect the depression-like behavioral responses to stresses. The results showed that the unfiltered mice presented significantly much more immobility in FST (Figure 2d), and less total active-social-interaction time (Figure 2e) than the filtered group. In summary, all of these suggested that the exposure of PM_2.5_ had the adverse effects on mood-related behaviors.

### 3.3. PM_2.5_ Exposure Impaired the Morphology of Hippocampus in Mice

**Figure 3:**
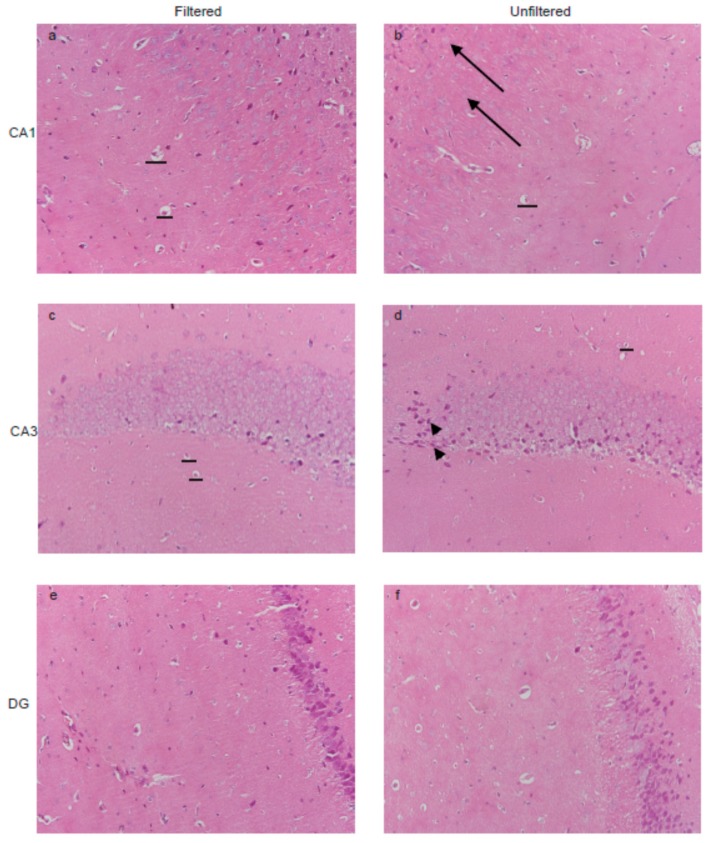
Effects of PM_2.5_ exposure on pathological changes. (**a**,**b**) PM_2.5_ exposure resulted in the degenerating neurons (no visible nucleolus) (arrowhead) in CA1 region; (**c**,**d**) The unfiltered mice showed the nuclear condensation and pyknosis in CA3 region; (**e**,**f**) The granule cells of DG region was decreased after PM_2.5_ exposure.

The hippocampus plays a prominent role in regulating mood-related behaviors, such as the depression-like and anxiety-like behavior [20], thus, we chose the hippocampus as the research object. Microphotographs of neuron structure in mice hippocampus are shown in Figure 3. In filtered mice, neurons in rat hippocampal CA1 and CA3 region organized orderly, and the dark stained nucleus were large. After PM_2.5_ exposure, the reduced pyramidal cells were observed (horizonta line). Meanwhile, some of the neuron cell nucleolus was invisible in CA1 region (arrow) in unfiltered mice. Moreover, the result also showed that the CA3 region displayed nuclear condensation and pyknosis (arrowhead), and the granule cells in DG region was decreased after PM_2.5_ exposure. These results indicate that exposed to PM_2.5_ impaired the neuron structure in mice hippocampus, which could cause mood-related behavior disorder.

### 3.4. PM_2.5_ Exposure Inhibited Hippocampal GR Activation in Mice

We compared the transcriptomes of the hippocampus in both groups by RNA-Seq. 2230 differentially expressed genes (DEGs) were identified, among them, 1405 were up regulated and 925 were down regulated. We analyzed the gene ontology (GO) annotation for the 2230 DEGs, to gain the clues of mechanisms related to the changed functions involved in the altered depression and anxiety behaviors. In the GO analysis results of biological process, the three items, including inflammatory response, response to wounding, and defense response were listed in the top six positions. It meant that the immune injury in the central nervous system of animal happened after PM_2.5_ exposure. Simultaneously, among these DEGs, behavior and locomotor behavior were ranked as the first and 4th by GO analyses, which was in well accordance with the results of depression and anxiety behavior tests (Supplement Table S1).

We also analyzed the KEGG (Kyoto Encyclopedia of Genes and Genomes) pathway. Among these DEGs, ribosome, cytokine-cytokine receptor interaction, hematopoietic cell lineage, Huntington’s disease, Parkinson’s disease, chemokine signaling pathway, and Alzheimer’s disease were ranked as the top seven changed categories by functional annotation (Supplement Table S2). Depression and anxiety are commonly seen in Parkinson’s disease and Alzheimer’s disease [21], and the typical symptom of Huntington’s disease was depression and anxiety [22].

**Figure 4:**
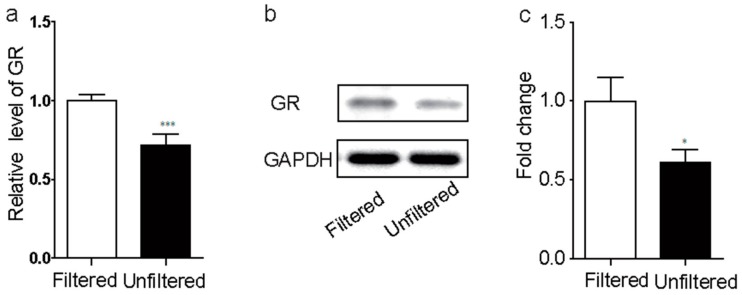
PM_2.5_ exposure inhibited hippocampal glucocorticoid receptors (GR) activation in mice. The GR expression level was decreased after PM_2.5_ exposure in both genes ((**a**), *p* < 0.001, t16 = 4.587) and protein ((**b**,**c**), *p* < 0.05, t14 = 2.269) level. * *p* < 0.05; *** *p* < 0.001.

Furthermore, glucocorticoid receptor in hippocampus negative feedback dampens the responses of hypothalamus-pituitary-adrenal (HPA) axis to stress [23,24]. Human studies have shown that a history of childhood abuse had decreased GR mRNA levels in the hippocampus, suggesting the decreased GR was associated with mental and mood disorder. We therefore investigated whether the increased glucocorticoid was regulated by glucocorticoid receptor in hippocampus. In the DEGs, we found the GR expression was significantly decreased by 1.36 folds in unfiltered group when compared with that in filtered group (Supplement Table S3). We further identify the levels of both GR mRNA by real-time PCR and GR protein by western-blot in the hippocampus, and found that both GR mRNA (Figure 4a) and GR protein (Figure 4b,c) was significantly decreased after exposure to PM_2.5._ Therefore, we speculate that the PM_2.5_ exposure could have the strong correlation with mental and mood disorder.

### 3.5. PM_2.5_ Exposure Induced Hippocampal Cytokine and Chemokine Activation in Mice

**Figure 5:**
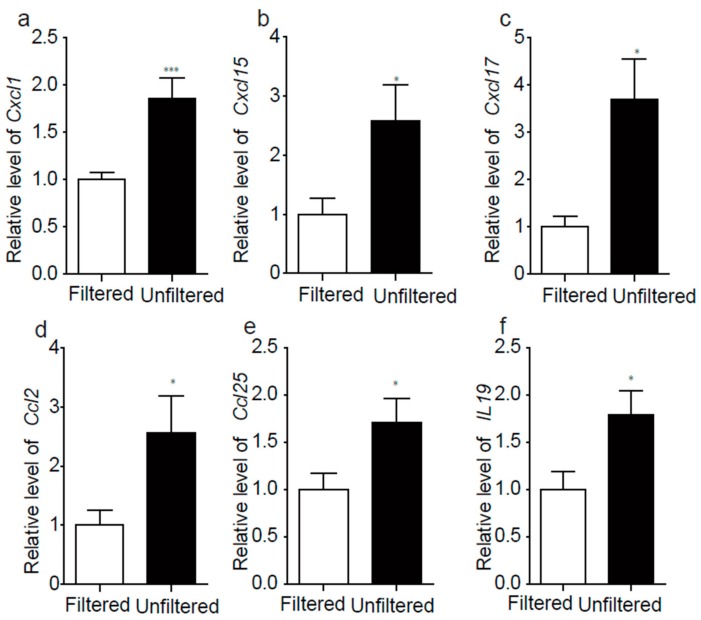
PM_2.5_ exposure induced hippocampal cytokine and chemokine activation in mice. The genes expression levels by real-time PCR. The Cxcl1 ((**a**), *p* < 0.01, t10 = 3.693), Cxcl15 ((**b**), *p* < 0.05, t10 = 2.355), Cxcl17 ((**c**), *p* < 0.05, t10 = 3.020), Ccl2 ((**d**), *p* < 0.05, t10 = 2.306), Ccl25 ((**e**), *p* < 0.05, t10 = 2.294), IL19 ((**f**), *p* < 0.05, t10 = 2.428). * *p* < 0.05; *** *p* < 0.001. PCR: Polymerase Chain Reaction.

PM_2.5_ exposure can result in the inflammation response, so we picked out some cytokine and chemokine and verified the expressions of them by real-time PCR. The results showed that the expression level of cytokine and chemokine were increased with exposure of PM_2.5_ (Figure 5, Supplement Table S3), which exhibited the same trend with the results of RNA sequencing.

## 4. Discussion

In recent years, epidemic mental illnesses were prevalent in China, but the etiology and pathogenesis of the disease remain largely unknown today. Our results displayed that the PM_2.5_ exposure promoted the secretion of GC in blood of both human and mice. The elevation of GC can exacerbate brain inflammation [12]. In the present study, the results of RNA-Seq and real-time PCR of the hippocampus showed that PM_2.5_ exposure up-regulated the expression of cytokine and chemokine. When combining with the increased concentration of GC, we suggested that the over secretion of GC activated immunity and inflammation in excess after PM_2.5_ exposure, which led to the series of mood-related behaviors disorder. For testing if the exposure of high concentration of PM_2.5_ can indeed obstruct the emotional expression, we did the systematically explored depression and anxiety-like behaviors, including OFT, L/DBT, EPMT, FST, and SIT, which cannot be tested in a human being. We further examined molecular processes that are involved in the pathophysiologic pathways by measuring RNA and protein biomarkers in hippocampus. All of these results support that exposure to heavy ambient air particles not only increases negative emotions, but also affect the normal molecular processes of mental health. Thus, we logically speculate that exposure to heavy particulate pollution has the possibility to aggravate the symptoms of mental illness, and may lead to mood-related behaviors disorder in human.

Under normal physiological conditions, the GR in hippocampus, via negative feedback inhibition, inhibits the activity of the HPA axis and the subsequent release of glucocorticoids from the adrenal cortex [25]. Under the condition of depression, the over secretion of GC after HPA being stimulated [14,15] can indirectly induce cell survival and reproduction by impairing the immune systems [26,27]. The GR in hippocampus has been found to be sensitive to early environmental cues and are involved in mood-related behaviors through a cortisol negative feedback loop. Consistent with this notion, we found that in our experiments, the expression of GR in hippocampus of mice in unfiltered group was decreased and the concentration of GC in plasma was increased, which was associated with an elevation in GC level. Moreover, the particles can enter into the brain area via the olfactory bulb nerve and act on the cognition related functions [28]. Based on the results, we may infer logically that the inhaled PM_2.5_ could trigger the decreased expression of GR in hippocampus, decreasing the negative feedback to HPA axis, and finally resulted in significant depression and anxiety-like behaviors failure.

Numerous studies reported the connection between mood disorder and activation of the innate immune responses. Exposure to PM_2.5_ is epidemiologically associated with immunity and inflammation [29,30,31]. Depression is associated with increased concentration of proinflammatory cytokines in circulation, such as IL-1β, IL-6, TNF-α, IFN-γ, and chemokines [32,33,34,35,36]. In the present study, we found that PM_2.5_ exposure up-regulated the cytokines and chemokines in hippocampus. Hippocampal inflammation is associated with changes in neuronal morphology and reduced neurogenesis, which can result in the cognitive dysfunction in dementia, epilepsy, and increased anxiety and depression-like responses [37,38,39]. Consistent with this, our KEGG results showed that PM_2.5_ exposure elevated the risk of Parkinson’s and Alzheimer’s diseases, indicating that the activation of inflammatory response system contributed to the mood-related behaviors disorder. When combining with the increased concentration of GC, we suggested that the over secretion of GC activated immunity and inflammation in excess, which led to the series of mood-related behaviors disorder.

## 5. Conclusions

**Figure 6:**
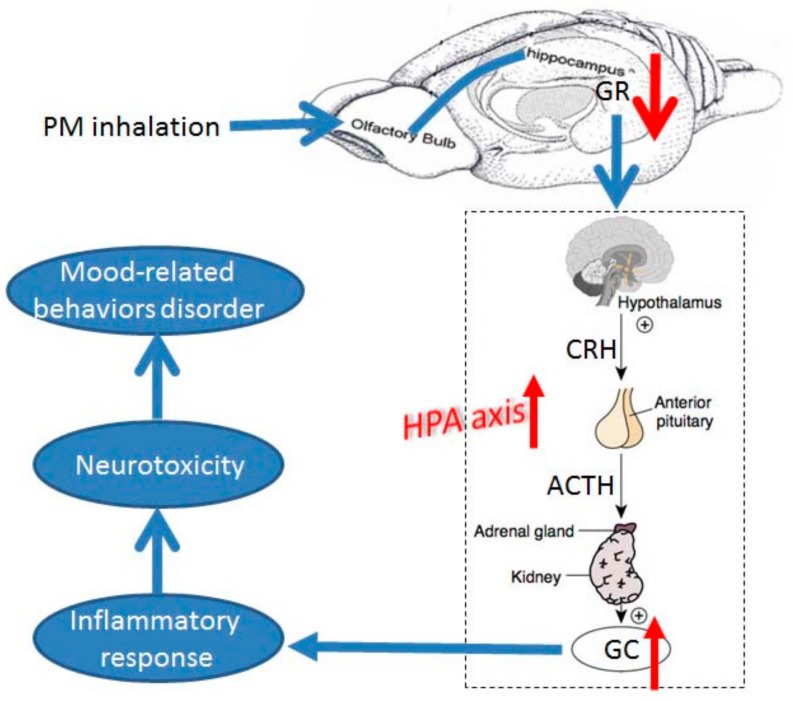
Mechanistic framework explaining how inhaled air pollutants disrupt mood related behavior. After PM_2.5_ exposure, the decreased expression level of GR in hippocampus result in the increased secreting of glucocorticoid, which activated inflammatory response of hippocampus, and thus provoked the mood-related behaviors disorder. PM: particulate matter; GR: glucocorticoid receptors; GC: glucocorticoid; HPA: hypothalamic-pituitary-adrenal; CRH: corticotropin-releasing hormone; ACTH: adrenocorticotropic hormone.

Using the comprehensive data from our experiments in combination with previously postulated mechanisms, we developed a mechanistic framework for future hypothesis testing (Figure 6). By the animal models, we found that there is a causal relationship between PM exposure and mental disorder. After PM_2.5_ exposure, the decreased expression level of GR in hippocampus resulted in the increased secreting of GC, which activated inflammatory response of hippocampus, and thus provoked the mood-related behaviors disorder. It should be note that the GC concentration in the blood of human subjects showed the same trend with mice. Thus, we have reason to speculate that exposure to heavy particulate pollution has the possibility to aggravate the symptoms of mental illness, and the risks of mood-related behaviors disorder in human. During the haze day (heavy pollution day), the susceptible subjects need to consider drug support.

These findings will add support to the urgent need to reduce air pollution exposure, given the growing burden of depression and anxiety in today’s highly polluted world. In some degree, our results remind that in the heavy pollution days, we need to pay more attention to the mental illness susceptible subjects.

## Supplementary Materials

The following are available online at www.mdpi.com/1660-4601/15/1/160/s1, Table S1: The gene ontology (GO) annotation, Table S2: The KEGG pathway, Table S3: The gene expression level of RNA-seq, Table S4: The primer for real-time PCR.

## Abbreviations

PM	Particulate Matter
ASD	Autism-Spectrum Disorders
HEPAF	High-Efficiency Particulate Air Filter
OFT	Open field test
EPMT	Elevated plus maze test
L/DBT	Light/Dark box test
FST	Forced swim test
SIT	Social interaction test
RNA-Seq	RNA-Sequence
HE	Hematoxylin and eosin
GC	Glucocorticoid
DEGs	Differentially Expressed Genes
GR	Glucocorticoid Receptor
HPA	Hypothalamus-Pituitary-Adrenal
PCR	Polymerase Chain Reaction
KEGG	Kyoto Encyclopedia of Genes and Genomes
BSA	Bovine Serum Albumin
TBS	Tris-buffered saline
CRH	Corticotropin-Releasing Hormone
ACTH	Adrenocorticotropic Hormone
