# Supplementary material for: Exposure to Ambient Air Particles Increases the Risk of Mental Disorder: Findings from a Natural Experiment in Beijing
Source: Int J Environ Res Public Health. 2018 Jan 19;15(1):160. doi: 10.3390/ijerph15010160 (PMC5800259; doi:10.3390/ijerph15010160)
Supplement: Supplementary file 1 [file ijerph-15-00160-s001.pdf]

International Journal of
Environmental Research and Public Health

MDPI

Supplementary Materials

Table S1. The gene ontology (GO) annotation.

Term	Count	%	*p*-value
GO: 0007610 ~ behavior	77	0.360133	1.28E-06
GO: 0006954 ~ inflammatory response	48	0.224498	7.81E-06
GO: 0009611 ~ response to wounding	66	0.308685	7.88E-06
GO: 0007626 ~ locomotory behavior	50	0.233852	8.65E-06
GO: 0042127 ~ regulation of cell proliferation	92	0.430289	1.11E-05
GO: 0006952 ~ defense response	79	0.369487	1.67E-05
GO: 0006412 ~ translation	60	0.280623	3.01E-05
GO: 0006811 ~ ion transport	112	0.52383	5.20E-05
GO: 0042330 ~ taxis	27	0.12628	8.54E-05
GO: 0006935 ~ chemotaxis	27	0.12628	8.54E-05
GO: 0007155 ~ cell adhesion	90	0.420934	1.55E-04
GO: 0009719 ~ response to endogenous stimulus	38	0.177728	1.60E-04
GO: 0006955 ~ immune response	78	0.36481	1.63E-04
GO: 0022610 ~ biological adhesion	90	0.420934	1.65E-04
GO: 0015672 ~ monovalent inorganic cation transport	55	0.257238	1.76E-04
GO: 0009725 ~ response to hormone stimulus	35	0.163697	1.78E-04
GO: 0006091 ~ generation of precursor metabolites and energy	47	0.219821	6.61E-04
GO: 0045637 ~ regulation of myeloid cell differentiation	15	0.070156	8.25E-04

Table S2. The KEGG pathway.

Term	Count	%	*p*-value
mmu03010: Ribosome	37	0.173051	6.71E-13
mmu04060: Cytokine-cytokine receptor interaction	55	0.257238	3.59E-07
mmu04640: Hematopoietic cell lineage	23	0.107572	8.80E-05
mmu05016: Huntington's disease	39	0.182405	9.02E-05
mmu05012: Parkinson's disease	30	0.140311	2.56E-04
mmu04062: Chemokine signaling pathway	34	0.15902	0.003003
mmu05010: Alzheimer's disease	34	0.15902	0.003003
mmu04512: ECM-receptor interaction	19	0.088864	0.003895
mmu00190: Oxidative phosphorylation	26	0.121603	0.004225
mmu04080: Neuroactive ligand-receptor interaction	44	0.20579	0.004707
mmu04010: MAPK signaling pathway	44	0.20579	0.006661
mmu04621: NOD-like receptor signaling pathway	15	0.070156	0.007143
mmu03050: Proteasome	12	0.056125	0.012419
mmu05014: Amyotrophic lateral sclerosis (ALS)	13	0.060802	0.021155
mmu04115: p53 signaling pathway	14	0.065479	0.039218
mmu03060: Protein export	4	0.018708	0.050367
mmu05322: Systemic lupus erythematosus	18	0.084187	0.062287
mmu04610: Complement and coagulation cascades	14	0.065479	0.06952
mmu04020: Calcium signaling pathway	29	0.135634	0.077435
mmu05414: Dilated cardiomyopathy	16	0.074833	0.083392
mmu05222: Small cell lung cancer	15	0.070156	0.086341
mmu05020: Prion diseases	8	0.037416	0.087794

KEGG: Kyoto Encyclopedia of Genes and Genomes

Table S3. The gene expression level of RNA-seq.

Gene Name	Log2 Ratio	*p*-value
GR	-0.454765608	0.042290687
Cxcl1	1.56010431	0.048813977
Cxcl15	1.984483417	0.009716695
Cxcl17	3.732245504	0.04431345
Ccl2	5.16715004	3.64E-17
Ccl25	0.86990332	0.012948782
Il19	4.68319874	0.01142824

GR: Glucocorticoid Receptors.

Table S4. The primer for real-time PCR.

Gene Name	Forward (5'-3')	Reverse (5'-3')
GR	CATACATGCAGGGTAGAGTCATTCTT	ACCAAGAGGTCCATGGTGTTT
Cxcl15	AACCTAGGCATCTTCGTCCG	TTCACCCATGGAGCATCAGG
Cxcl17	TGCAAAGATTGGTTCCTGCAA	TCCTGTGGTGCTTTTGGTGT
Ccl2	CACTCACCTGCTGCTACTCA	GCTTGGTGACAAAAACTACAGC
Ccl25	AGTTCACTGATCCCATAGGCA	GGTTTAAGGGGGCCACCAAT
IL19	CTCCTGGGCATGACGTTGATT	GCATGGCTCTCTTGATCTCGT
Gapdh	CCCTTAAGAGGGATGCTGCC	TACGGCCAAATCCGTTCACA

PCR: Polymerase Chain Reaction.
